# 4D label-free proteomics analysis of oxygen-induced retinopathy with or without anti-VEGF treatment

**DOI:** 10.1186/s12864-024-10340-z

**Published:** 2024-04-26

**Authors:** Zhaokai Xu, Yubo Wu, Jianbo Mao, Yiqi Chen, Huan Chen, Shian Zhang, Jiafeng Yu, Xinyi Deng, Lijun Shen

**Affiliations:** 1 State Key Laboratory of Ophthalmology, Optometry and Visual Science, Eye Hospital, Wenzhou Medical University, Wenzhou, Zhejiang Province China; 2 Department of Ophthalmology, Zhejiang Provincial People’s Hospital (Affiliated People’s Hospital, Hangzhou Medical College), Hangzhou, China

**Keywords:** Proteomic, 4D label-free, Oxygen-induced retinopathy, Retinopathy of prematurity, Anti-VEGF therapy

## Abstract

Oxygen-induced retinopathy (OIR) animal model is widely used for retinopathy of prematurity (ROP) researches. The purpose of this study was to identify proteins and related pathways of OIR with or without anti-vascular endothelial growth factor (VEGF) treatment, for use as biomarkers in diagnosing and treating ROP. Nine samples were subjected to proteomic analysis. Retina specimens were collected from 3 OIR mice, 3 OIR mice with anti-VEGF treatment and 3 normal mice (control group). Liquid chromatography-tandem mass spectrometry analysis was performed using the 4D label-free technique. Statistically significant differentially expressed proteins, gene ontology (GO) terms, Kyoto Encyclopedia of Genes and Genomes (KEGG) pathway representations, InterPro (IPR) and protein interactions were analyzed. In total, 4585 unique proteins were identified as differentially expressed proteins (DEPs). Enrichment analysis of the GO and KEGG indicated functional clusters related to peptide biosynthetic and metabolic process, cellular macromolecule biosynthetic process and nucleic acid binding in OIR group. For anti-VEGF treatment group, DEPs were clustered in DNA replication, PI3K/Akt signaling pathway and Jak/STAT signaling pathway. Proteomic profiling is useful for the exploration of molecular mechanisms of OIR and mechanisms of anti-VEGF treatment. These findings may be useful for identification of novel biomarkers for ROP pathogenesis and treatment.

**Supplementary Information:**

The online version contains supplementary material available at 10.1186/s12864-024-10340-z.

## Introduction

Preterm birth is a significant global health problem. With the decreasing total fertility rate, which is most pronounced in developing countries, a growing number of studies have focused on the development and quality of life of preterm infants [1, 2]. Further, retinopathy of prematurity (ROP), with an overall estimated incidence of 50,000 infants annually, is a leading cause of childhood blindness in industrialized countries [3].

To ablate the peripheral avascular retina and reverse neovascularization, the gold standard treatment of ROP has been laser photocoagulation [4]. On the other hand, laser therapy might cause visual impairment, such as peripheral visual field defect and refractive error [5]. Because neovascularization in ROP is largely vascular endothelial growth factor (VEGF) driven and anti-VEGF therapy appears to have fewer ocular side effects, various intravitreal anti-VEGF medications have substituted or been in conjunction with traditional laser therapy [6, 7].

Because of the special physiological condition of premature infants, animal models are widely used in ROP researches. The mouse oxygen-induced retinopathy (OIR) model is a well-established animal model for ROP researches, which is characterized by retinal vessel change from hyperoxia-induced vessel loss to neovascular in normoxia [8, 9]. OIR model is analyzed and studied mainly in angiogenesis disease field, but some researches have also reported neuronal damage and pathological effects on retina in OIR model [10]. Furthermore, VEGF plays an extremely important role in OIR model, as well as pathogenesis of preterm infants. Because of multiple interactions among vascular, neuronal, glial, and immune cells in retinal vascular pathology, it is necessary to conduct researches in whole retinal tissue to gain more information about the mechanism of pathogenesis.

Proteomics has been widely used in ophthalmic research and proved to be reliable. Because of the advancement in the mass spectrometry technology, proteomics could facilitate accurately identification of potential disease markers for subsequent diagnosis and prognosis, as verified by many previous studies [11, 12]. Therefore, label-free quantitative proteomics strategy was applied to comprehensively characterize the retinal proteome in a model of VEGF-induced retinal insult as well as to evaluate the efficacy of anti-VEGF treatment.

In this study, we identified proteins and related pathways examined in OIR model and examined effects of anti-VEGF treatment. There is a possibility for revealing alterations in the major metabolic functions, cellular stress, and corresponding dysfunctions, because of oxygen-mediated insult and protective characteristics of anti-VEGF in retina.

## Results

### Analysis of significantly differentially expressed proteins

**Fig. 1:**
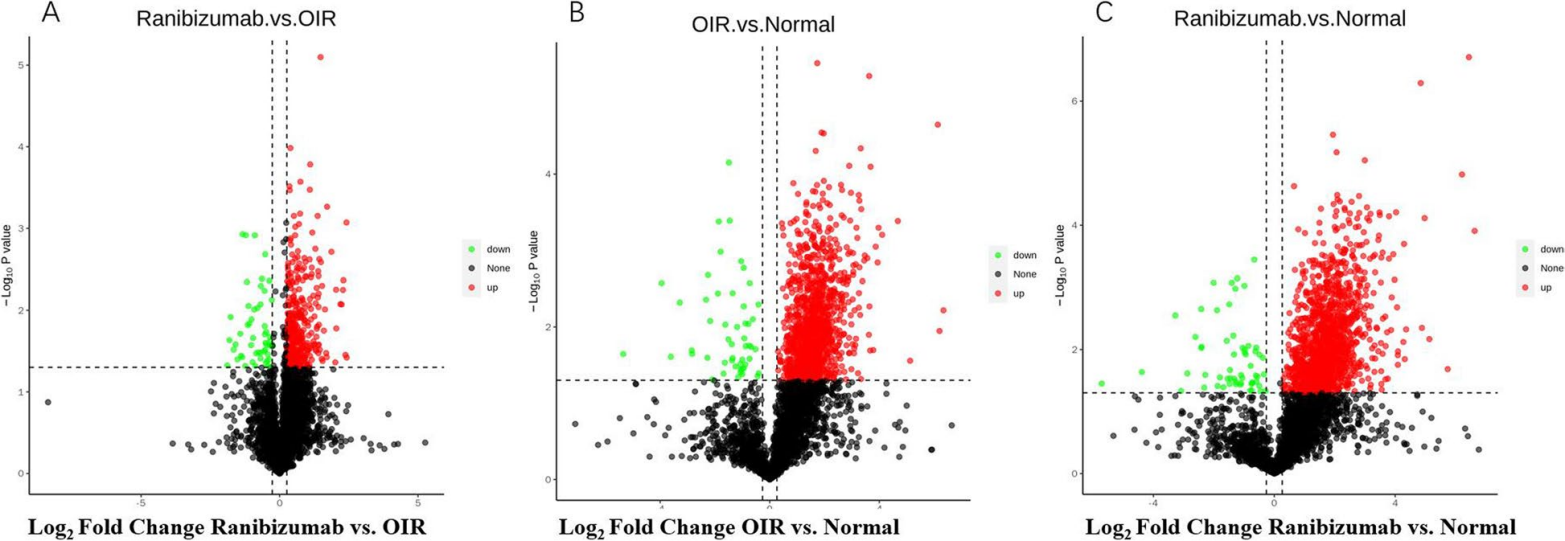
Volcano plot of differentially expressed proteins. **A** Comparisons of the volcano plot of the differential proteins expressed between the ranibizumab group and the OIR group. **B** Comparisons of the volcano plot of the differential proteins expressed between the OIR group and the normal group. **C** Comparisons of the volcano plot of the differential proteins expressed between the ranibizumab group and the normal group. The X-axis represents protein difference (log2-transformed fold changes), and the Y-axis represents the corresponding − log10-transformed *P* values. Red dots indicate significantly up-regulated proteins, green dots denote significantly down-regulated proteins, and gray dots symbolize proteins with no significant change

**Fig. 2:**
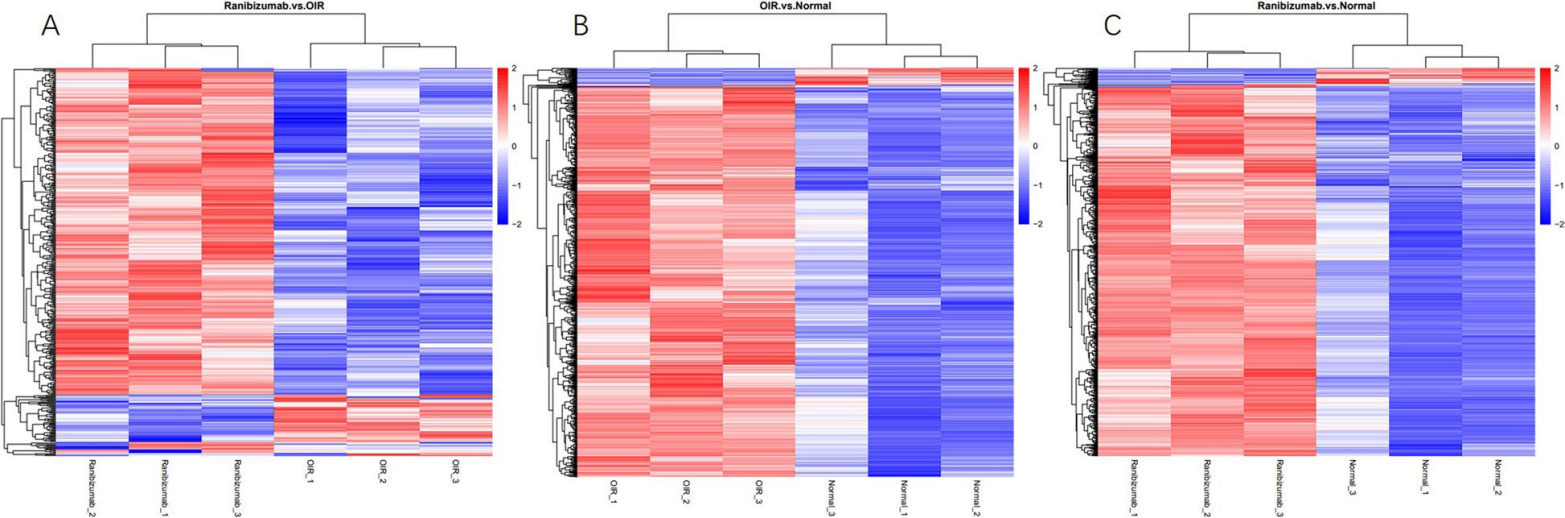
Comparisons of the heatmaps of the differential proteins expressed in three groups. **A** Comparisons of the heatmaps of the differential proteins expressed between the ranibizumab group and the OIR group. **B** Comparisons of the heatmaps of the differential proteins expressed between the OIR group and the normal group. **C** Comparisons of the heatmaps of the differential proteins expressed between the ranibizumab group and the normal group. Higher red and blue intensities indicate higher degrees of upregulation and downregulation, respectively

**Fig. 3:**
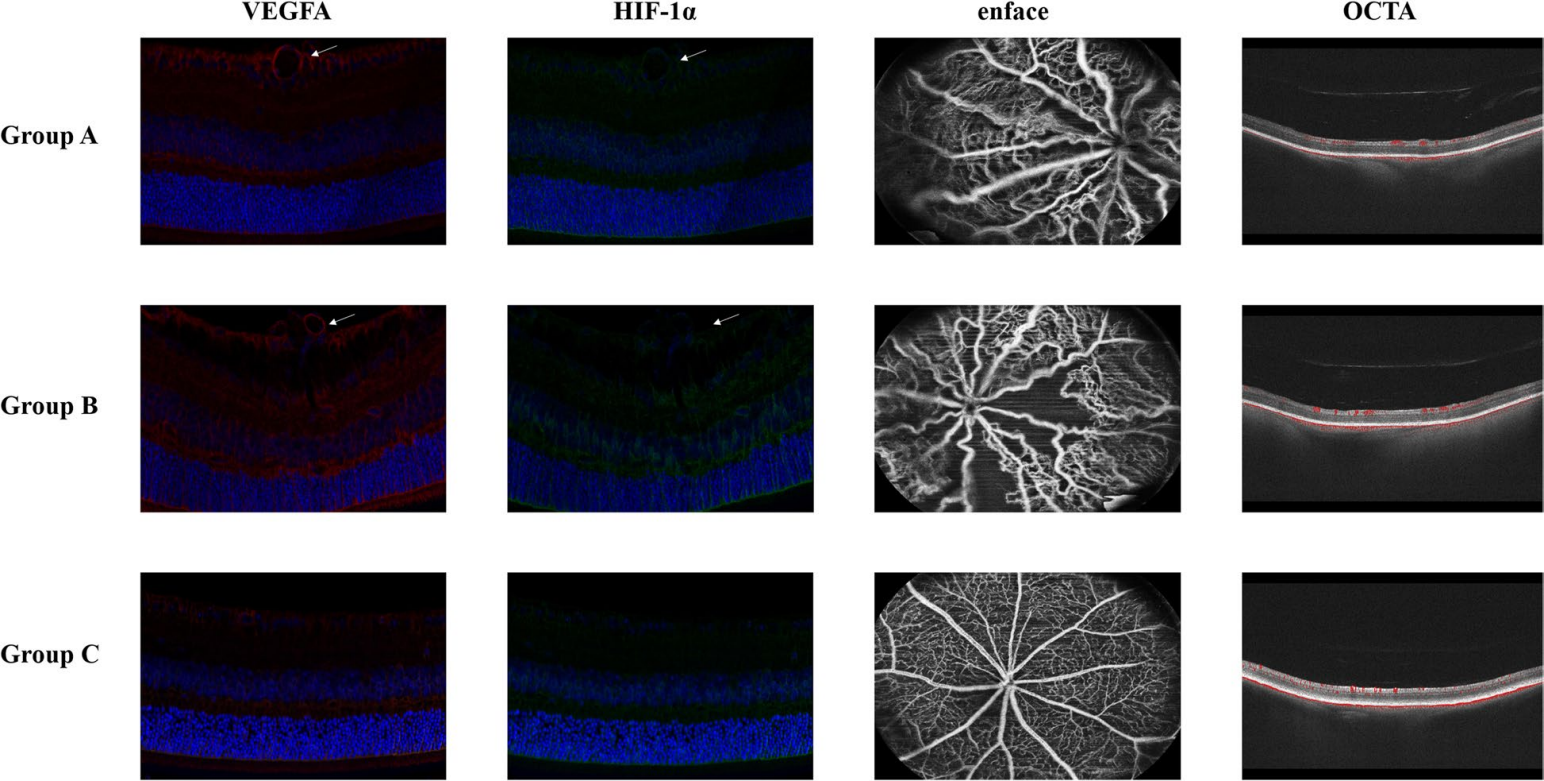
VEGFA and HIF-1α double-stained confocal images of retinas and OCTA images in three groups. Group A: the ranibizumab group; Group B: the OIR group; Group C: the normal group. At P17, neovascularization emerges on the surface of the retina (arrows). Avascular area and tortuous neovascularization could be directly observed on the enface images. Abnormal vascularity also caused inhomogeneous distribution of blood flow signals on OCTA images

For the details of the data, 159,155 matched spectra, 35,450 peptides, 4585 identified and quantifiable proteins were obtained from 9 samples. The number of up-regulated proteins and down-regulated proteins was listed in the Table 1 and Fig. 1. Hierarchical clustering analysis was performed on dysregulated proteins of three groups, and the heatmaps generated from the analysis showed a clear distinction in protein expression levels among the ranibizumab, OIR and normal groups (Fig. 2). Retinal images of immunohistochemistry and OCTA was present in Fig. 3.

**Table 1:** Number of differentially expressed proteins

Compared samples	Num. of total quant	Regulated type	FC > 1.2	FC > 1.3	FC > 1.5	FC > 2.0
Group A vs. Group B	4329	up-regulated	371	309	198	81
		down-regulated	56	48	34	21
Group B vs. Group C	4019	up-regulated	1465	1458	1439	1284
		down-regulated	68	65	61	45
Group A vs. Group C	3976	up-regulated	1236	1232	1217	1080
		down-regulated	57	57	53	37

Group A: the ranibizumab group, Group B: the OIR group, Group C: the normal group

*Num. of Total Quant.* Number of total quantifiable proteins, *FC* Fold-change

### GO functional analysis of the DEPs

**Fig. 4:**
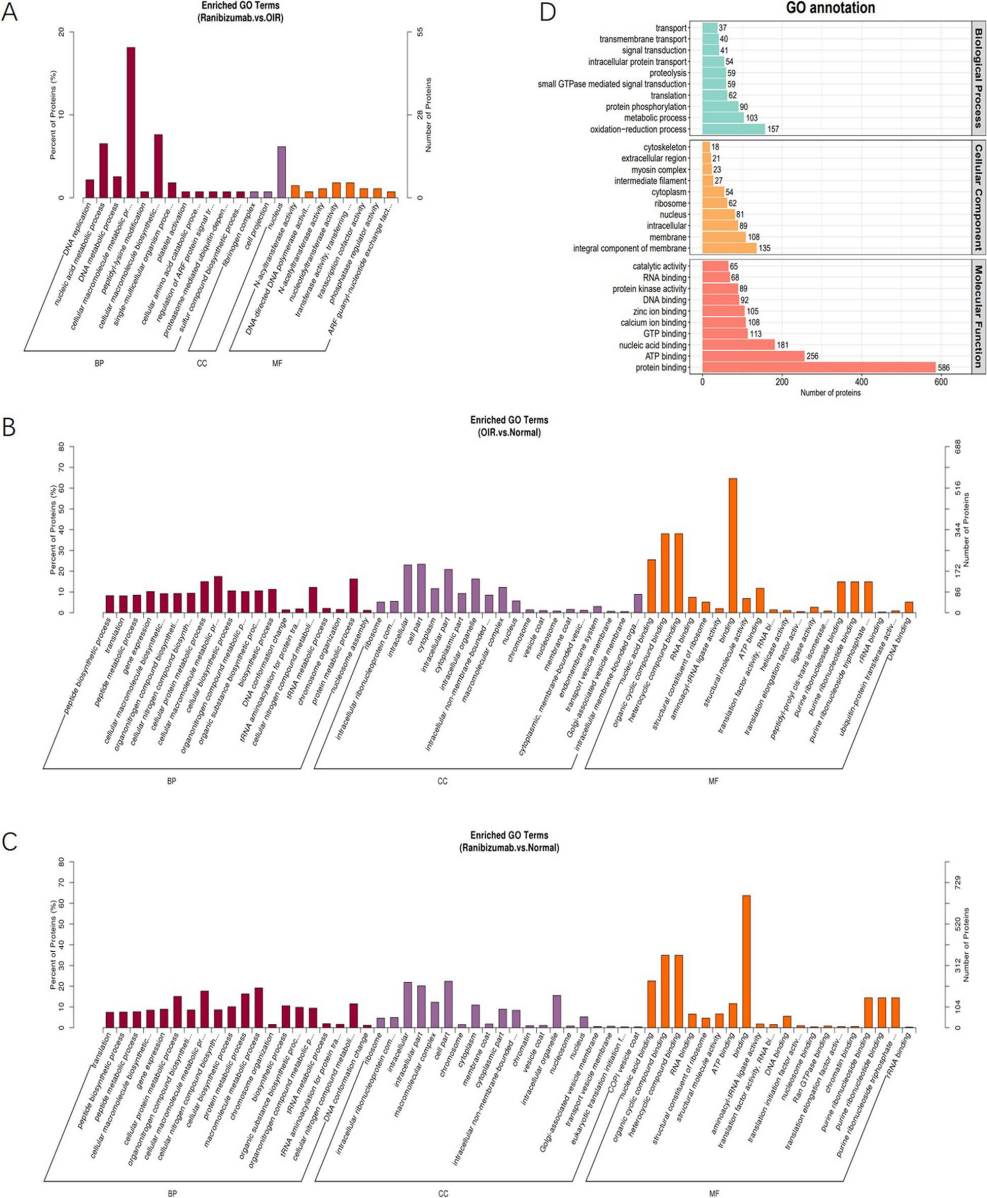
GO annotation and GO enrichment analyses of the DEPs. **A** GO enrichment analysis of the DEPs (Top 20) between the ranibizumab group and the OIR group. **B** GO enrichment analysis of the DEPs (Top 20) between the OIR group and the normal group. **C** GO enrichment analysis of the DEPs (Top 20) between the ranibizumab group and the normal group. **D** GO annotation analysis of the DEPs (Top 10) of the three groups

We conducted GO analysis to enrich and cluster the differentially expressed proteins (DEPs) of the ranibizumab, OIR, and normal groups. Details of the cellular components (CC), molecular functions (MF), and biological processes (BP) are elaborated in Fig. 4. GO annotation analysis suggested that the DEPs of the three groups mainly participate in MF, such as protein binding, ATP binding, nucleic acid binding, and GTP binding. In BP, oxidation − reduction process, metabolic process and protein phosphorylation had many DEPs aggregation. Using GO annotation-enriched analysis, it indicated how anti-VEGF affects vascular changes. The GO enrichment of the ranibizumab group was involved in regulation of response to cellular macromolecule metabolic process, nucleic acid metabolic process and cellular macromolecule biosynthetic process (*p* < 0.05). On the other hand, the GO enrichment of the OIR group showed that a large number of up-regulated DEPs were enriched in terms related to MF, such as organic cyclic compound binding, heterocyclic compound binding, nucleic acid binding, et al. (*p* < 0.05). The GO enrichment analysis of the ranibizumab group was similar with the OIR group when compared with normal group.

### KEGG pathway analysis functional analysis of the DEPs

**Fig. 5:**
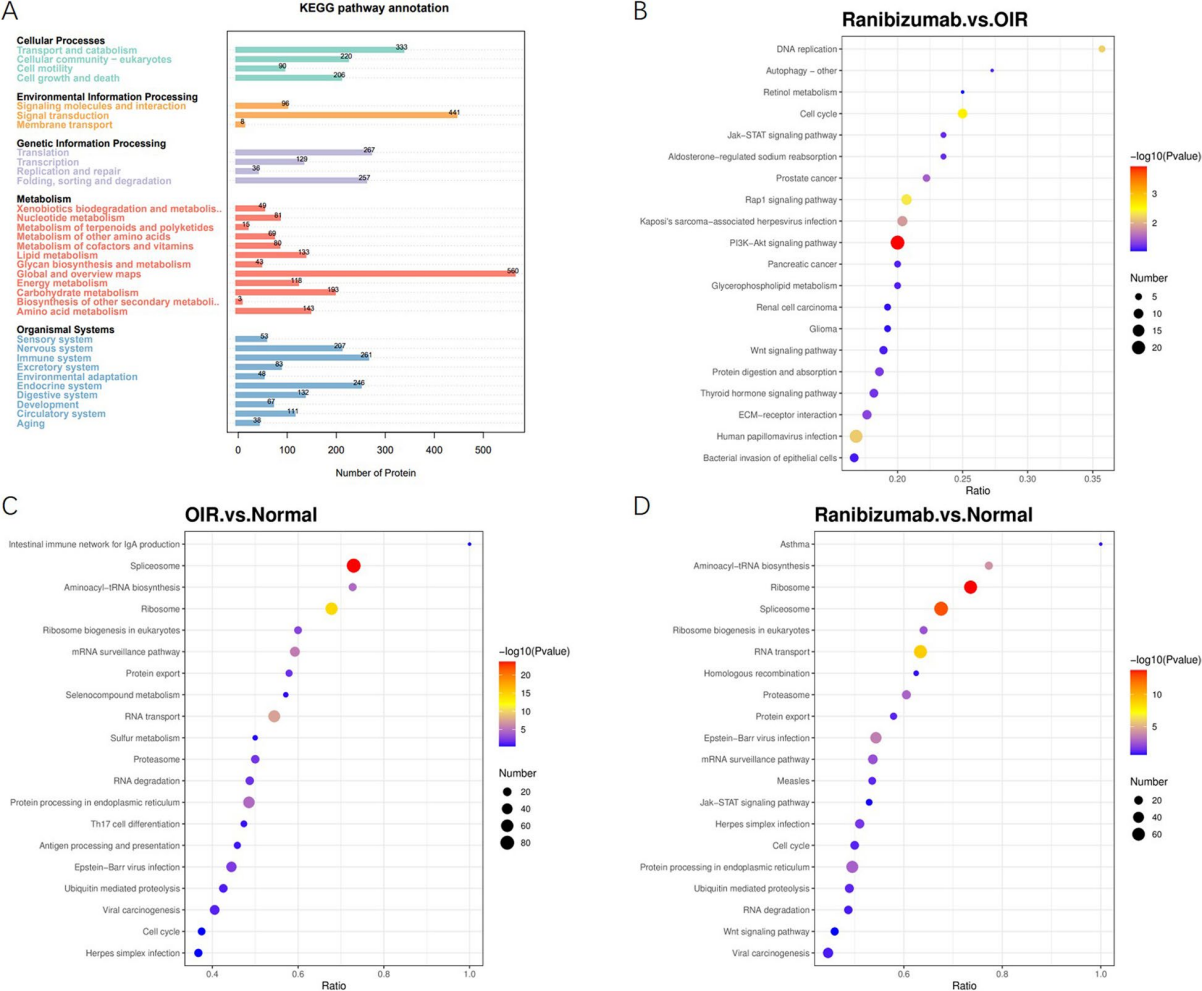
KEGG analyses of the DEPs. **A** KEGG annotation of number of DEPs of the three groups. **B-D** KEGG pathway enrichment bubble plot

For further physiological characterization of samples from different groups, we conducted annotation and enrichment analysis of KEGG pathway to identify the main biochemical metabolic pathways involving the differential metabolites. In total, all differential metabolites of three groups were enriched in 34 pathways (Fig. 5). KEGG pathway analysis revealed that the most obvious pathway affected by intravitreal injection of ranibizumab was “PI3K/Akt signaling pathway”. A large number of DEPs were detected in this pathway.

For OIR group, spliceosome, ribosome and other pathway associated with RNA-transport had significant difference compared to control group. The KEGG analysis of ranibizumab. vs. normal group presented similar result.

### IPR enrichment analysis of the DEPs

**Fig. 6:**
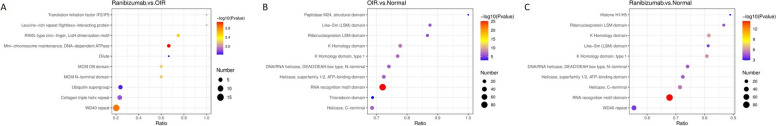
IPR analyses of the DEPs. **A** IPR analyses of the DEPs between the ranibizumab group and the OIR group. **B** IPR analyses of the DEPs between the OIR group and the normal group. **C** IPR analyses of the DEPs between the ranibizumab group and the normal group

We conducted IPR analysis to enrich and cluster the DEPs of the ranibizumab, OIR, and normal groups (Fig. 6). IPR enrichment analysis showed that 21 significantly enriched functional domains were identified between the ranibizumab and OIR groups, especially mini-chromosome maintenance (MCM), DNA-dependent ATPase and WD40 repeat domain. On the other hand, 46 domains were identified between the OIR and normal groups. The most significant enriched functional domains were RNA recognition motif domain, K homology domain (KHD) and C-terminal domain (CTD). The IPR analysis of ranibizumab. vs. normal group presented similar result.

### PPI analysis of the DEPs

**Fig. 7:**
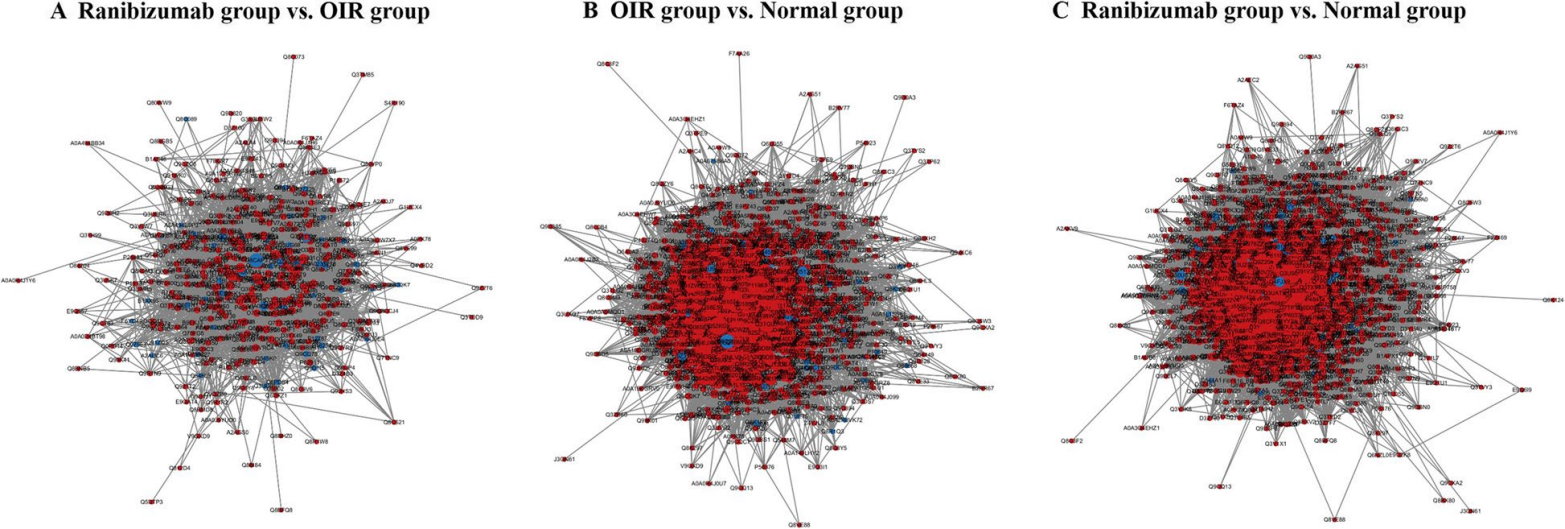
PPI analysis of the DEPs. **A** DEPs interaction networks of the DEPs between the ranibizumab group and the OIR group. **B** DEPs interaction networks of the DEPs between the OIR group and the normal group. **C** DEPs interaction networks of the DEPs between the ranibizumab group and the normal group. Red node: up-regulated. Blue node: down-regulated

Using the STRING online database and Cytoscape software, we established a protein–protein interaction network of DEPs (Fig. 7). In comparison of the ranibizumab and OIR group, the network consisted of 411 nodes and 5273 edges (ranibizumab group vs. OIR group). Compared with the OIR group, DEPs up-regulated in the ranibizumab group were mainly enriched in pathways related to cell responses, migration and growth, transcription of DNA, GTP-binding, nucleotide-binding, protein homeostasis, neuronal migration, and promotes neurogenesis. DEPs down-regulated in the ranibizumab group were enriched in pathways related to nucleotide excision repair, modulation of proteasomal degradation, mRNA processing and splicing, and tRNA modification.

In comparison of the OIR and normal group, the interaction network of DEPs contained 1274 nodes and 88,453 edges. Compared with the normal group, DEPs up-regulated in the OIR group were mainly enriched in pathways related to correct protein translation and folding, heat shock protein formation, protein sumoylation, RNA helicase, and folding of actin and tubulin. DEPs down-regulated in the OIR group were enriched in pathways related to cell protection against oxidative stress, neuroprotective mechanisms, cell growth and transformation, glutathione biosynthesis, and protein phosphorylating. In comparison of the ranibizumab and normal group, the interaction network of DEPs contained 1515 nodes and 112,066 edges. The result of regulation was similar with comparison of the OIR and normal group.

**Fig. 8:**
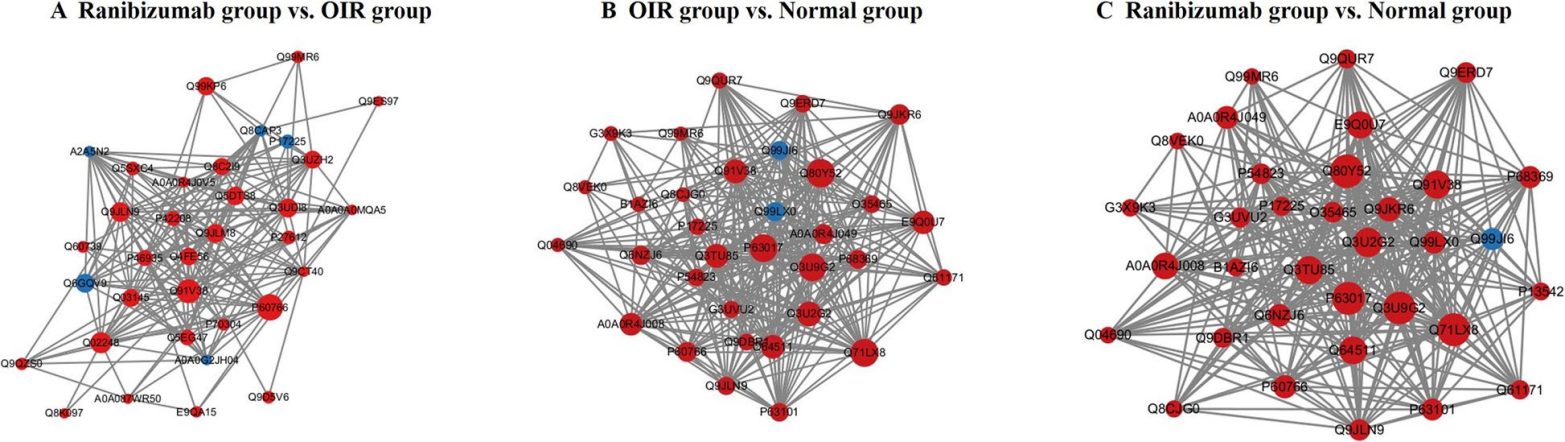
Simplified interactome map of PPI analysis. **A** DEPs interaction networks of the DEPs between the ranibizumab group and the OIR group. **B** DEPs interaction networks of the DEPs between the OIR group and the normal group. **C** DEPs interaction networks of the DEPs between the ranibizumab group and the normal group. Red node: up-regulated. Blue node: down-regulated

Another smaller-scale simplified interactome map was present in Fig. 8. The nodes with high scores were selected for display.

## Discussion

This study implemented 4D label-free proteomics and analyses of functional protein network and pathways to elaborate the global and specific proteomics alterations in the OIR and anti-VEGF treatment animal models. Because of the characteristic of ROP and physiological structure of infants, it’s extremely difficult to obtain pathological eye contents, such as aqueous humor, vitreous humor, or retinal tissue [13]. Nevertheless, full-fledged OIR model could maturely simulate pathological mechanism of ROP, which is characterized by abnormal avascular area and neovascularization. Up to now, some proteomics studies have been conducted on OIR model [14–16]. Although there were few researches focus on the influence caused of treatment on the model. At the same time, anti-VEGF treatment takes on increasing importance in retinal ischemic diseases [17, 18]. In this study, we introduced anti-VEGF treatment to OIR model for proteomics analysis. We thought that revelation of the complex proteome changes happening after anti-VEGF treatment would be an important source for detecting ROP-specific biological targets. And it will help to find biomarkers for diagnosis and disease treatment, and for the better knowledge of the pathogenic mechanisms of the disease.

### Neovascularization in OIR

ROP is constituted by two oxygen-dependent stage. The first stage is induced by the hyperoxic environment and activated immediately after the premature birth; the increase of oxygen initiates a decrease in VEGF, which causes the stagnation of retinal vascular development. At this stage, the retinal vessels are so vulnerable and ineffective that retina becomes hypoxic [19]. When oxygen concentration recovers to a normal level, the second stage starts with an increase in the levels of VEGF, which promotes an excessive growth of abnormal retinal vessels that extend into the vitreous and even retinal disease [20]. When the mouse is born, its retinal development is not yet mature, and the hyperoxic environment would further inhibit retinal vascular development [21]. After the pups return to normal room air environment, aberrant retinal vessels further evolve to the formation of neovascular tufts [22]. The pathological mechanism of OIR model is extremely similar to human ROP [23]. The retinal angiogenic changes under hypoxia are stimulated by up-regulated VEGF [24], while the Müller cells release VEGF for vessel growth in the deep vascular plexus [25].

In this study, we used OCTA to visualize retinal vascular structure. Compared with control group, abnormal avascular zone and neovascularization area could be clearly observed in OIR mice.

The result of proteomic analysis displayed that a lot of proteins participated in pathological angiogenesis. Heat shock proteins (HSPs) were significantly upregulated proteins, which plays an important role in angiogenesis [26]. The expression of HSPB1 is increased in DR and cause retinal injury by enhancing VEGF expression [27, 28]. HSPC could induce retinal neovascularization by regulating HIF-1 and VEGF [29]. α-crystallins, which are small heat shock proteins, were detected to be expressed severely. Their expression is dramatically upregulated in numerous retinal diseases, such as mechanical injury, ischemic insults, age-related macular degeneration (AMD), and DR [30].

Erythropoietin (EPO) production can be stimulated by hypoxia, and have a direct relationship, and both are stimulated by HIF-1 [31]. EPO have promotional functions in vascular proliferation and endothelial cell proliferation [32]. We also detected a significantly differential protein, U4/U6.U5 tri-snRNP-associated protein 1, which is one of the building blocks of the spliceosome and plays a role in hypoxia-induced regulation of EPO gene expression [33].

### Neuronal tissue in OIR

The results revealed that in addition to the changes detected on proteins responding to hypoxia and inducing pathological neovascularization, some changes in protein expression took place in the neuronal tissue of the retina. As previous researches showed, not only angiogenesis, but also neurodevelopment was affected by abnormal oxygen environment [16]. The effect of hyperoxia-hypoxia induction is harmful to the retinal neurons due to the presence of oxidative stress [34].

ROP can also be considered one of the neurodegeneration diseases. The hypoxic damage could induce the production of free radicals, inadequate blood supply and other inflammatory actions, along with the apoptotic effect in neuronal cells [35]. It has been reported that persistent ectopic synapses, prolonged cellular apoptosis, and gliosis exist in the OIR retina [36], which is similar with ROP pathogenesis. Many children with a history of ROP show persistent vision impairment, and there is evidence of an association between ROP and neurosensory disabilities [37]. A study shows that preterm infants with severe ROP have a significantly poorer outcome at 11 years than preterm infants without ROP, with neurosensory impairments detected in 50% of the infants who had suffered from severe ROP [38]. We found that OIR showed an up-regulated expression of 14–3-3 proteins, which have proved the importance of the 14–3-3 protein family in the development of the nervous system [39].

Glial cells participate in neuroinflammation and synaptic homeostasis, the latter being essential for maintaining the physiological function of the central nervous system (CNS) [40]. Retinal microglia have been noted to be involved in the OIR model while their role in the OIR process is still unclear. In mouse OIR model, the number of retinal microglia was increased and associated with a loss of deep retinal vessels [10]. In addition, it has been observed that microglia are the predominant myeloid cells in the neovascularization area in mouse OIR [41]. Further study showed that microglia might cooperate with astrocytes, promote the regrowth of blood vessels after vascular occlusion in OIR, and thus result in less neovascularization afterward in the mouse OIR model [42]. In our study, we detected some proteins associated with the differentiation and metabolism of glial cell. Up-regulated expression of these proteins corresponded to the activity of glial cells in OIR. These mediators might play a potential protective role in the inflammatory and apoptotic conditions of ischemic injury.

### Proteins involved in ncRNAs in OIR

GO analysis demonstrated that peptide biosynthetic and metabolic process, cellular macromolecule biosynthetic process and nucleic acid binding had the major role in significant enrichment. KEGG enrichment analysis indicated that a large number of differential proteins were mainly expressed in upregulation and involved in non-coding RNAs (ncRNAs), including spliceosome, RNA transport, rRNA processing and mRNA surveillance. NcRNAs are functional RNAs that are not translated into proteins and regulate various retinal diseases [43]. Based on their molecular weight, they are classified into microRNAs (miRNAs), long non-coding RNAs (lncRNAs), and circular RNAs (circRNAs) [44]. In existing research, miRNAs are the most extensively explored ncRNAs about ROP. MiR-18a-5p and miR-145, which are up-regulated in mouse OIR model, could enhance pathological neovascularization [45, 46]. Some studies have uncovered the effect of partial lncRNAs in ROP, which alleviates retinal neovascularization by dampening the Akt/VEGF pathway [47–49]. Some circRNAs in ROP have been reported to regulating retinal neovascularization [50, 51]. In this study, we detected a large number of proteins that are involved in ncRNA regulation of OIR. We identified some DEPs encoded by serine/arginine-rich splicing factors (SRSF) family. SRSF family plays an essential role in the progression of neurodevelopment [52], and has been reported in mouse OIR model [53]. In addition, we detected some of small nuclear ribonucleoproteins (snRNPs). It has been proved that snRNP contributes ischemia-hypoxia regulation [54]. RNA-binding proteins (RBPs) were also significantly differential proteins detected in this study. RBPs are critical effectors of gene expression, and as such their malfunction could induce many diseases [55]. It has been proved that RBPs also participate in diabetic retinopathy (DR) and affect retinal neovascularization through binding to VEGF and increasing its stability [56–58]. Protein splicing factors play key roles in mediating the progression of the spliceosome pathway [59], and we identified plenty of differential splicing factor subunits expressed significant differently in OIR model.

### Proliferation and repairment after anti-VEGF treatment

Via OCTA image, we observed that eyes taking anti-VEGF injection had less abnormal retinal area, comparing to those without treatment. As shown in previous animal studies and clinical trials, intravitreal injection of anti-VEGF displayed a beneficial effect on reducing neovascular area and promoting vessels growth toward the peripheral retina after treatment [60, 61].

GO enrichment analysis demonstrated that among the cellular macromolecule and nucleic acid metabolic process showing strong positive correlations with the ranibizumab group, DNA replication and its correlative metabolites displayed the most significant enrichment. The IPR enrichment analysis showed that a large number of proteins about MCM was detected in the ranibizumab group, including MCM3, MCM4, MCM5, MCM7 and their fragments. Past research has shown that MCM played a necessary role in the biologic processes and pathways of DNA replication [62, 63], DNA repair [64], cell cycle [65, 66], et al. MCM5 has been prove to be an indispensable role in the zebrafish retina for ensuring efficient genomic duplication and cell-cycle progression [67].

KEGG enrichment analysis indicated that Jak/STAT and HIF-1 were major down-regulated signaling pathways expressed in treatment group. It has been reported that activated STAT3 to lead to neovascularization by activation of NADPH oxidase [68]. And HIF-1 is well-known as a stimulus that leads to an increase in VEGF and angiogenesis [69]. EPO could also be stimulated by HIF-1 in a hypoxia environment [31], and promote vascular proliferation. From the view of result, intravitreal injection of anti-VEGF could control abnormal angiogenesis by these pathways.

Among the up-regulated proteins, we detected a great deal of collagens and fibronectins enriched in PI3K/Akt signaling pathway. It might be related to degeneration of neovascularization. The studies have shown that collagen and laminin-entactin enhances the stability of vessels [70]. In addition, low concentrations of type IV collagen promote elongation, and high concentrations of type IV collagen stabilize microcapillaries [71].

## Conclusion

In conclusion, our study revealed that the proteomic characteristics of retinal tissue in mouse OIR model were significantly different from the proteomic characteristics of the control group. Further analysis between the ranibizumab and OIR group indicated partial signaling pathways and DEPs were affected by intravitreal injection of anti-VEGF. These findings may be useful for identification of novel biomarkers for ROP pathogenesis and treatment.

## Material and methods

### Animals

**Fig. 9:**
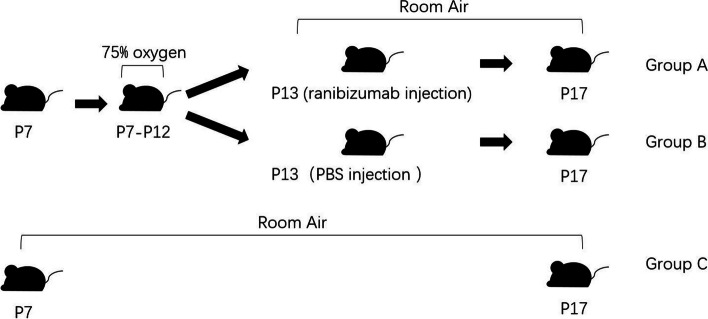
Outline of the animal model

In this study, SPF C57BL/6 J mice (purchased from Hangzhou hangsi Biotechnology Co., Ltd. (Hangzhou, China)) were housed under standard conditions with 12-h dark/12-h light cycle and fed with standard laboratory pellets and water ad libitum. The OIR model was generated as described in previous study [8]. To sum up, the newborn mice at postnatal day 7 (P7) and their nursing mothers were exposed to 75% ± 1% oxygen environment for 5 days until P12. After that, they were returned to normal room air to induce OIR. On the other hand, OIR mice were divided into two groups. Intraocular injections of 5 μg /0.5 µL of ranibizumab into the vitreous body (group A) were performed at P13 using 33-gauge needles (Hamilton, Bonaduz, Switzerland). Group B didn’t take any therapeutic intervention but intraocular injections of 0.5µL phosphate-buffered saline (PBS; Proteintech Inc, USA). Moreover, another group of normal newborn mice (control group, group C) were housed under normal room air conditions. All mice were humanely euthanized with an intraperitoneal injection of saline-diluted pentobarbital sodium (200 mg/kg) and eyeballs collected at P17 (late hypoxic phase and the peak of neovascularization) to assess the effect of OIR and anti-VEGF treatment on retinal proteome. The study design is described in Fig. 9. In previous study, it demonstrated that postnatal weight would affect outcome in the OIR model [72]. In this study, only the pups weighing between 6.3 and 7.5 g at P17 were included in the study.

### OCTA Examination

Optical coherence tomography angiography (OCTA) examination of the fundus in each group of mice was performed using Beiming-Kun (400,000 ultrawide-angle, full-field sweep OCT) from Toward Pi (Beijing) Medical Technology Ltd. (Beijing, China). All pups were pharmacologically dilated after receiving intraperitoneal anesthesia (1.25% tribromoethanol) according to the manufacturer’s operating instructions to capture high resolution scan imaging images of the various retinal layers.

We picked enrolled mice by OCTA images. For ranibizumab and OIR group, the inclusion criteria are abnormal fundus vascular, including pathological avascular area and neovascularization. After OCTA examination, pups were decapitated and eyes enucleated.

### Proteomics

Detailed methods of protein extraction, trypsin digestion, HPLC fractionation, modification enrichment and LC–MS analysis were described in Supplementary Data.

### Immunohistochemistry

Enucleated eyes were fixed with 4% paraformaldehyde (PFA) for 1 h, and processed for paraffin embedding. Five-micrometer thick sections were subjected to antigen retrieval (20xsodium citrate antigen retrieval solution, pH6.0), blocked and incubated either with HIF-1α antibody (66,730–1-Ig; 1:50; Proteintech, Rosemont, IL, USA) or VEGFA polyclonal antibody (19,003–1-ap; 1:200; Proteintech, Rosemont, IL, USA) followed by horseradish peroxidase (HRP) conjugated secondary antibodies. Samples were imaged confocal laser microscope (KF-PRO-120; KFBIO; Ningbo, China).

### Statistical analysis

All data were analyzed by SPSS 25.0 software (IBM, Chicago, IL, USA). The normal distribution of the data was confirmed by the Shapiro‒Wilk and Kolmogorov–Smirnov tests. T test and Mann–Whitney U test were used to compare differences between two groups. The differences at *p* < 0.05 were considered statistically significant.

### Supplementary Information

**Supplementary Material 1.**

## Data Availability

The mass spectrometry proteomics data have been deposited to the ProteomeXchange Consortium (http://proteomecentral.proteomexchange.org) via the iProX partner repository with the dataset identifier PXD045378.
