# Supplementary material for: 4D label-free proteomics analysis of oxygen-induced retinopathy with or without anti-VEGF treatment
Source: BMC Genomics. 2024 Apr 26;25:415. doi: 10.1186/s12864-024-10340-z (PMC11046906; doi:10.1186/s12864-024-10340-z)
Supplement: Supplementary file 1 — Supplementary Material 1. [file 12864_2024_10340_MOESM1_ESM.docx]

**Supplementary Data**

**Proteomics**

**Chemicals and Materials.** Acetonitrile (ACN), formic acid (FA), and water (UHPLC-MS grade) were all purchased from Thermo Fisher Scientific (Waltham, MA, USA). Triethylammonium bicarbonate buffer 1M (TEAB), iodoacetamide (IAA), chloroacetamide (CAA), Tris(2-carboxyethyl) phosphine (TCEB), and ammonium bicarbonate (ABC) were all purchased from Sigma Aldrich Corp. (St. Louis, MO, USA). Sodium dodecyl sulfate (SDS), acetone, and urea were all purchased from Sinopharm Group (Beijing, China). Trypsin (Mass Spectrometry Grade) was purchased from Promega (Madison, WI, USA).

**Sample Preparation.** Sample was ground individually in liquid nitrogen and lysed with SDT lysis buffer (containing 100Mm Nacl) and 1/100 volume of DTT, followed by 5 min of ultrasonication on ice. After reacting at 95℃ for 8-15 min and ice-bath for 2min, the lysate was centrifuged at 12000 g for 15 min at 4℃. And the supernatant was alkylated with sufficient IAM for 1 h at room temperature in the dark. Then samples were completely mixed with 4 times volume of precooled acetone by vortexing and incubated at -20℃ for at least 2 h. Samples were then centrifuged at 12000 g for 15 min at 4℃ and the precipitation was collected. After washing with 1mL cold acetone, the pellet was dissolved by Dissolution Buffer (DB buffer).

**Protein Quality Test.** BSA standard protein solution was prepared according to the instructions of Bradford protein quantitative kit, with gradient concentration ranged from 0 to 0.5 g/L. BSA standard protein solutions and sample solutions with different dilution multiples were added into 96-well plate to fill up the volume to 20 µL, respectively. Each gradient was repeated three times. The plate was added 180 μL G250 dye solution quickly and placed at room temperature for 5 minutes, the absorbance at 595 nm was detected. The standard curve was drawn with the absorbance of standard protein solution and the protein concentration of the sample was calculated. 20 μg of the protein sample was loaded to 12% SDS-PAGE gel electrophoresis, wherein the concentrated gel was performed at 80 V for 20 min, and the separation gel was performed at 120 V for 90 min. The gel was stained by Coomassie brilliant blue R-250 and decolored until the bands were visualized clearly.

**Trypsin Treatment.** Each protein sample was taken and the volume was made up to 100 μL with DB lysis buffer (8 M Urea, 100 mM TEAB, pH 8.5), trypsin and 100 mM TEAB buffer were added, sample was mixed and digested at 37 ° C for 4 h. Then trypsin and CaCl2 were added digested overnight. Formic acid was mixed with digested sample, adjusted pH under 3, and centrifuged at 12000 g for 5 min at room temperature. The supernatant was slowly loaded to the C18 desalting column, washed with washing buffer (0.1% formic acid, 3% acetonitrile) 3 times, then added elution buffer (0.1% formic acid, 70% acetonitrile). The eluents of each sample were collected and lyophilized.

**LC-MS/MS Analysis.** UHPLC-MS/MS analyses were performed using a nanoElute UHPLC system (Bruker, Germany) coupled with a tims TOF pro2 mass spectrometer (Bruker, Germany) in Novogene Co., Ltd. (Beijing, China). Firstly, mobile phase A (100% water, 0.1% formic acid) and B solution (100% acetonitrile, 0.1% formic acid) were prepared. The lyophilized powder was dissolved in 10 μL of solution A, centrifuged at 14,000 g for 20 min at 4 ℃, and 200 ng of the supernatant was injected into the Liquid chromatography-mass spectrometry system to detect. The model type of the UHPLC was nanoElute with nano-upgraded, and the analytical column was a home-made analytical column (15 cm×100 μm, 1.9 μm). The elution conditions of liquid chromatography were shown in **Table S1**. The tims TOF pro2 mass spectrometry with Captive Spray ion source. spray voltage was set to 2.1 kV. The full scan range of the mass was from m/z 100 to 1700 and the Ramp time was 100 ms. The Lock Duty Cycle was set to 100%. The settings of PASEF were as following: 10 MS/MS scan (a total cycle time of 1.17 sec), ionic strength threshold of 2500, scheduling target intensity of 20000. The raw data of MS detection was named as “.d”.

**Proteome Functional Analysis.** The all-resulting spectra were searched against UniProt database by the search engines: MaxQuant (Bruker, Tims). The search parameters of Proteome Discoverer are set as follows: mass tolerance for precursor ion was 10 ppm and mass tolerance for product ion was 0.02 Da. Carbamidomethyl was specified as fixed modifications, Oxidation of methionine (M) was specified as dynamic modification, and loss of methionine at the N-Terminal. A maximum of 2 missed cleavage sites were allowed. The search parameters of MaxQuant are set as follows: mass tolerance for precursor ion was 20 ppm and mass tolerance for product ion was 0.05 Da. Carbamidomethyl was specified as fixed modifications, Oxidation of methionine (M) was specified as dynamic modification, and acetylation was specified as N-Terminal modification. A maximum of 2 missed cleavage sites were allowed. In order to improve the quality of analysis results, the software MaxQuant further filtered the retrieval results: Peptide Spectrum Matches (PSMs) with a credibility of more than 99% was identified PSMs. The identified protein contains at least 1 unique peptide. The identified PSMs and protein were retained and performed with FDR no more than 1.0%. The protein quantitation results were statistically analyzed by T-test. The proteins whose quantitation significantly different between experimental and normal groups, (p < 0.05 and FC> 1.2 [fold change, FC]), were defined as differentially expressed proteins (DEP).

Gene Ontology (GO) and InterPro (IPR) functional analysis were conducted using the interproscan program against the non-redundant protein database (including Pfam, PRINTS, ProDom, SMART, ProSite, PANTHER), and the databases of COG (Clusters of Orthologous Groups) and KEGG (Kyoto Encyclopedia of Genes and Genomes) were used to analyze the protein family and pathway. DEPs were used for Volcanic map analysis, cluster heat map analysis and enrichment analysis of GO, IPR and KEGG. The probable protein-protein interactions were predicted using the STRING-db server (<http://string.embl.de/>) and Cytoscape software.

**Table S1.** nanoElute Liquid chromatography elution gradient table

| Time (min) | Flow rate (mL/min) | Mobile phase A (%) | Mobile phase B (%) |
| --- | --- | --- | --- |
| 0 | 300 | 98 | 2 |
| 45 | 300 | 78 | 22 |
| 50 | 300 | 65 | 35 |
| 55 | 300 | 20 | 80 |
| 60 | 300 | 20 | 80 |
